# Uncovering Necroptosis in Alzheimer’s Disease: A Systematic Review of Evidence Across Experimental Models

**DOI:** 10.1007/s10571-025-01601-w

**Published:** 2025-10-03

**Authors:** Nishi Shah, Gopal Natesan, Richa Gupta

**Affiliations:** 1 Department of Pharmacology, Institute of Pharmacy, Nirma University, Sarkhej-Gandhinagar Hwy, Gota, Ahmedabad, Gujarat 382481 India; 2 Department of Pharmaceutical Chemistry, Institute of Pharmacy, Nirma University, Sarkhej-Gandhinagar Hwy, Gota, Ahmedabad, Gujarat 382481 India

**Keywords:** Alzheimer’s disease, Necroptosis, Programmed cell death, Amyloid-β, RIPK1, RIPK3, MLKL

## Abstract

Alzheimer's disease (AD), one of the most challenging neurodegenerative disorders, with high prevalence worldwide, is characterized by progressive cognitive decline and accumulation of amyloid-β plaques and neurofibrillary tau tangles. Despite significant research, the limited efficacy of current treatments underscores the critical need to identify novel pathogenic mechanisms and therapeutic targets. Necroptosis, a regulated and highly inflammatory form of programmed cell death, has emerged as one of the key contributors to AD pathogenesis. This systematic review comprises 25 high-quality in vivo, in vitro, and autopsy studies, published between 2015 and 2025, extracted from PubMed, Scopus, and Science Direct databases. The keywords include "necroptosis", "RIPK1", "RIPK3", "MLKL", "pMLKL", "necroptosis inhibitors", "Alzheimer’s disease", and "neurodegeneration". The review summarizes the multiple molecular mechanisms, including TNF-α/TNFR1 signaling, TRIF-mediated RIPK3 activation, and RHIM-dependent MLKL phosphorylation, associated with necroptosis in the pathogenesis of AD. All the studies converge on necroptosis as a central pathogenic pathway linking key molecular and cellular abnormalities observed in AD. The accumulated evidence strongly supports prioritizing the development of brain-penetrant necroptosis inhibitors and clinical validation of associated biomarkers. These insights signal a significant shift in AD therapeutics, moving from symptomatic treatment to mechanistically targeted interventions that can alter disease progression.

## Introduction

Alzheimer’s disease, the most prevalent form of dementia, represents a growing global public health challenge with significant medical, social, and economic implications. Clinically, AD is characterized by a progressive deterioration in memory, cognition, language, and functional capacity, with early manifestations often including subtle symptoms such as forgetfulness, impaired judgment, and difficulties with planning or spatial orientation (Al-Kuraishy et al. 2025a; Livingston et al. 2024). More than 55 million individuals globally are living with dementia, with AD accounting for approximately 60–70% of cases (Gustavsson et al. 2023; Zhang et al. 2025). This figure is projected to rise to 78 million by 2030 and 139 million by 2050, driven largely by aging populations, particularly in low and middle-income countries, where over 60% of individuals with dementia currently reside (Chen et al. 2024a, b; Thapa et al. 2024). The economic burden of AD is equally substantial, with global costs expected to surpass $1 trillion annually by 2050, much of which stems from informal caregiving (Mahboob et al. 2024). In the United States, approximately 6.9 million adults aged 65 years and older were living with AD in 2024, a number anticipated to rise to 13.8 million by 2060 in the absence of effective preventive or disease-modifying therapies (Conic et al. 2025). Additionally, AD ranks as the fifth leading cause of death among older adults in the U.S., accounting for more than 119,000 deaths in 2021, a 141% increase since 2000 (Mobaderi et al. 2024).These statistics underscore the urgent need for the broad implementation of preventive strategies to mitigate the growing human and economic toll of Alzheimer’s disease.

The pathophysiological landscape of AD is governed by a multifactorial interplay of molecular and cellular disruptions that collectively lead to progressive neurodegeneration (Jiang et al. 2025a, b). One of the earliest and most salient features is the deposition of extracellular beta-amyloid (Aβ) plaques, which are the product of the aberrant cleavage of amyloid precursor protein (APP) by β-secretase (BACE1) and γ-secretase (Al-Kuraishy et al. 2025b, c). These neurotoxic aggregates disrupt synaptic function, trigger innate immune responses, and set off a cascade of chronic neuroinflammation (Azargoonjahromi 2024). Neuroinflammation further amplifies this degenerative cascade. Initially, a protective mechanism, sustained activation of microglia and astrocytes in the presence of Aβ and tau aggregates, results in the persistent release of pro-inflammatory mediators such as tumor necrosis factor-α, interleukin-1, and interleukin-6, which contribute to neuronal damage and synaptic loss (Dias et al. 2025). Hyperphosphorylated tau, another major pathological hallmark, dissociates from microtubules and forms intracellular neurofibrillary tangles (NFTs), disrupting axonal transport and cytoskeletal stability (Abubakar et al. 2025; Alsubaie et al. 2022; Jiang et al. 2025a, b). The spread of pathological tau through prion-like mechanisms further exacerbates neuronal damage and is closely associated with the progression of cognitive decline (Pandey et al. 2025). Moreover, oxidative stress and mitochondrial dysfunction also play pivotal roles in the disease progression. Mitochondrial dysfunction fails to meet the high-energy demands of neurons, rendering them increasingly susceptible to apoptotic and necrotic processes (Singh and Dilawari 2025). In addition to mitochondrial failure, dysregulation of calcium homeostasis, endoplasmic reticulum stress, and defective autophagy-lysosomal pathways contribute to neuronal injury in AD (Angst et al. 2025). Genetic influences further modulate the risk and onset of the disease. Mutations in APP, presenilin 1 (PSEN1), and presenilin 2 (PSEN2) are associated with early onset familial AD, which accounts for approximately 10% and is characterized by excessive Aβ production, while the apolipoprotein ε4 (APOE ε4) allele remains the most significant genetic risk factor for late-onset AD, accounting for about 90% of cases, which is primarily linked to impaired Aβ clearance and is strongly associated with aging (Al-Kuraishy et al. 2025b; Nan et al. 2025). Besides genetic factors, environmental and lifestyle factors such as exposure to neurotoxicants, poor diet, physical inactivity, and disrupted sleep patterns also influence disease susceptibility and progression. The initiation of complex pathological processes in Alzheimer’s disease often precedes clinical symptoms by years or even decades, emphasizing the critical need for early detection and therapeutic strategies that effectively target the molecular drivers of disease progression (Woźniak et al. 2024).

Neuronal cell death is a hallmark feature of AD, and for many years, this degeneration was predominantly attributed to classical mechanisms of cell death, namely, apoptosis and necrosis (Homma et al. 2024). Apoptosis, a genetically programmed and energy-dependent process, plays a critical role in maintaining cellular homeostasis by eliminating damaged or dysfunctional cells in a highly controlled and non-inflammatory manner (Ekundayo et al. 2024). Morphologically, it is characterized by cell shrinkage, chromatin condensation, DNA fragmentation, and membrane blebbing, ultimately resulting in phagocytic clearance without triggering an immune response (Guo et al. 2024). In contrast, necrosis represents an uncontrolled, passive form of cell death usually induced by severe cellular stress or injury, such as oxidative damage, excitotoxicity, or energy failure (Zhang and Niu 2024). Necrotic cells typically undergo rapid swelling, membrane rupture, and subsequent release of intracellular contents into the surrounding tissue, provoking a robust inflammatory response (Homma et al. 2024). While both mechanisms have been observed in AD, they fail to fully explain the complex patterns of neuronal loss and neuroinflammation characteristic of the disease. Consequently, recent research has shifted toward exploring newly identified, regulated forms of programmed cell death, including necroptosis, ferroptosis, pyroptosis, parthanatos, oxeiptosis, cuproptosis, and alkaliptosis (Yao Chen et al. 2024a, b).

Among these, necroptosis, a programmed cell death, is typically characterized by both necroptosis and apoptosis, and is triggered when apoptotic pathways are blocked, which leads to the activation of key molecules involved in necroptosis, is RIPK1, RIPK3, and MLKL (Yuan and Ofengeim 2024). In contrast to apoptosis, which does not start an immune response, necroptosis is linked to disruption of plasma membranes and consequent release of damage-associated molecular patterns like ATP, HMGB1, and mitochondrial DNA, leading to a robust inflammatory response (Nakano et al. 2022). The process starts with the help of death receptors like TNF, Fas, and TLRs. When these receptors are activated and apoptosis is inhibited, generally due to caspase-8 blockage, RIPK1 interacts with RIPK3 to form the necrosome, which further activates MLKL, leading to cell membrane disruption and necroptotic cell death (Seo et al. 2021). Necroptosis has also been implicated in various diseases like ischemic stroke, myocardial infarction, inflammatory bowel disease, retinal degeneration, infectious diseases, and certain cancers, where it amplifies tissue damage through uncontrolled inflammation (Dai et al. 2021). Emerging evidence has also implicated necroptosis in the progression of multiple neurodegenerative diseases such as AD, Parkinson’s disease, Amyotrophic lateral sclerosis, and Multiple sclerosis, causing neuronal damage and death (Thadathil et al. 2021). These results collectively implicate necroptosis as a key contributor to neuronal death and neuroinflammation in various neurological disorders.

**Fig. 1:**
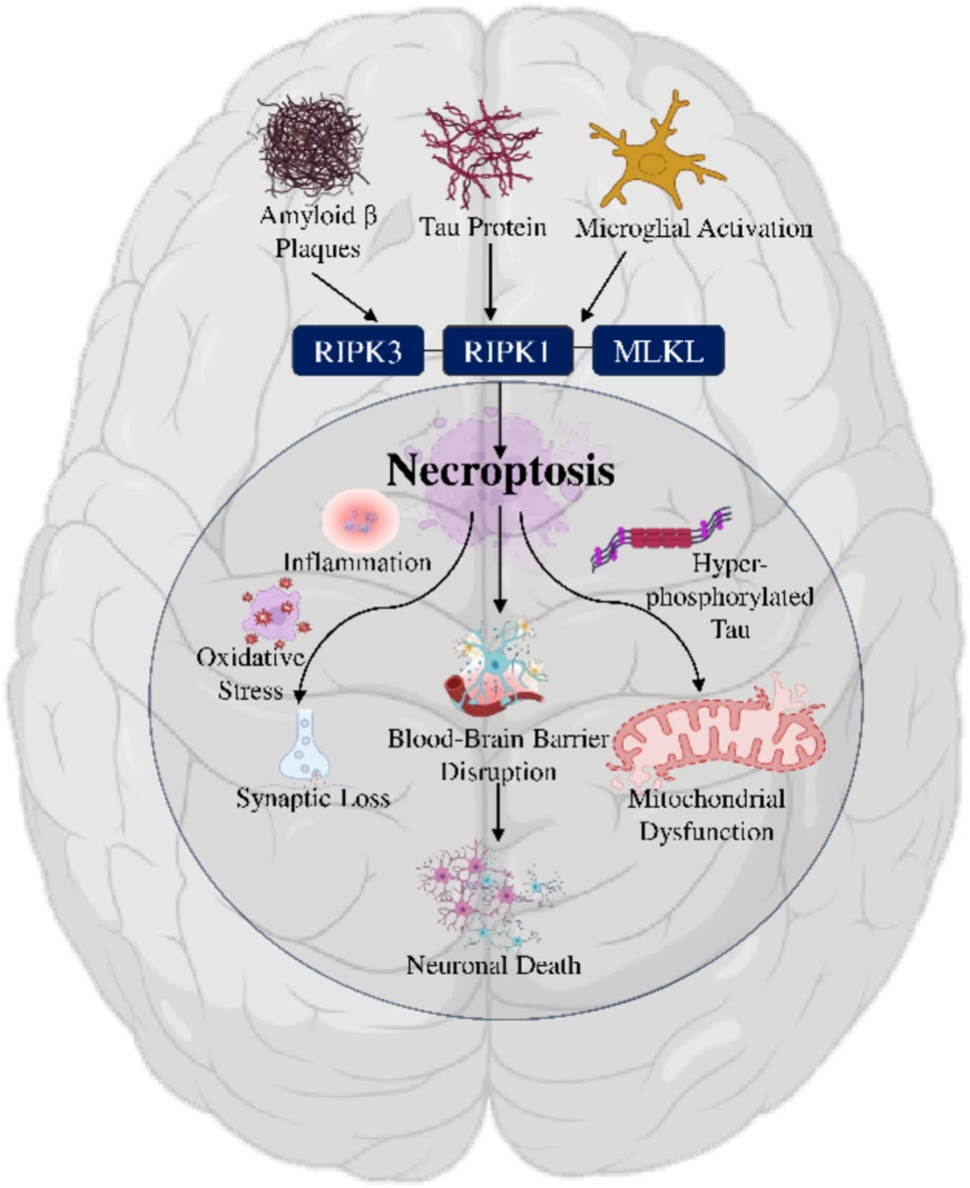
Schematic Representation of the Role of Necroptosis in Alzheimer’s Disease

In AD, increasing evidence supports a role for necroptosis in neuronal death and neuroinflammation. Elevated levels of RIPK1, RIPK3, and phosphorylated MLKL have been identified in postmortem AD brains, linking necroptosis with disease severity (Zhang et al. 2023). Amyloid-β and tau aggregates appear to trigger this pathway via oxidative stress, mitochondrial dysfunction, and microglial activation, while DAMP release sustains microglial activation in a vicious inflammatory cycle (Choi et al. 2023). Emerging research illuminates how necroptosis is intricately linked with key AD pathologies amyloid‑β aggregation, tau hyperphosphorylation, oxidative stress, and neuroinflammation, though these interactions remain under‑explored. Aβ oligomers (Aβo), rather than insoluble fibrils, have been shown to activate necroptosis via TNF-α/TNFR1 signaling in microglia, triggering RIPK1/RIPK3/MLKL activation in neurons (Salvadores et al. 2022). Intriguingly, the RIPK1–RIPK3 necrosome itself forms amyloid-like fibril structures that enhance cell surface expression of APP processing products (sAβPPβ), promoting further Aβ plaque formation (Zhang et al. 2023). Hyperphosphorylated tau is also a direct activator of necroptosis. Studies in AD mouse models and cell culture reveal that pTau induces RIPK1/RIPK3/MLKL activation and concurrently stimulates NF-κB-driven pro-inflammatory cytokine production, which further amplifies microglial activation and neuronal damage (Zhang et al. 2023). Further, oxidative stress and mitochondrial dysfunction both contribute to and are amplified by necroptosis. Mitochondrial damage induced by necroptosis raises ROS levels, reinforcing mitochondrial dysfunction and promoting neuron vulnerability. Elevated ROS may also facilitate RIPK1 activation by damaging redox-sensitive inhibitory pathways (Richard and Mousa 2022). Moreover, necroptosis actively drives neuroinflammation. DAMPs released during necroptotic neuronal death (e.g., HMGB1, ATP) activate microglia toward a pro-inflammatory M1 phenotype (Kaur et al. 2023). In AD models, inhibition of RIPK1 signaling reduces M1 microglial markers and inflammatory cytokines, promoting microglial phenotypic shift toward the anti-inflammatory, Aβ-clearing M2 state (Zhang et al. 2023). Thus, inhibition of necroptosis offers a promising therapeutic approach, with antagonists such as necrostatin-1 and RIPK1 inhibitors currently being explored for their neuroprotective effects (Kang et al. 2025). Understanding these interactions in an integrated manner would not only deepen insight into AD pathogenesis but also enhance the precision of targeted therapies (Fig. 1).

Figure 1 depicts the role of necroptosis in Alzheimer’s disease pathogenesis. It illustrates how core AD pathologies like amyloid-beta plaques, tau protein, and microglial activation converge to activate RIPK1, RIPK3, and MLKL, initiating necroptosis. This regulated cell death further drives a vicious cycle of neurodegeneration, contributing to inflammation, oxidative stress, blood–brain barrier disruption, synaptic loss, mitochondrial dysfunction, and increased hyperphosphorylated tau, culminating in widespread neuronal death. This systematic review examines the growing evidences and emerging role of necroptosis in the pathogenesis of AD. The review compiles findings from both preclinical and clinical studies that establish links between necroptosis and hallmark AD pathologies, including amyloid-β accumulation, tau hyperphosphorylation, microglial activation, and blood–brain barrier disruption. It further explores the initiation and execution of the necroptotic pathway, different pathway involves in necroptosis leading to neurodegeneration, detailing the role of MLKL as the final executor of cell death, and examining how necroptosis contributes to AD-related neurodegeneration. Additionally, the review discusses current efforts to modulate necroptosis through pharmacological agents and genetic interventions, as well as their clinical development status. Despite growing interest in necroptosis as a neurodegenerative mechanism, a comprehensive synthesis of evidence linking this pathway specifically to AD is still lacking. By consolidating these insights across experimental models and human data, the review aims to clarify the mechanistic underpinnings of necroptosis in AD and various translational potential as a therapeutic target. By addressing those gap, this review contributes to a deeper understanding of neurodegeneration and helps guide future research and therapeutic innovation.

## Methodology

### Search Strategy and Databases

A comprehensive literature search was conducted across multiple electronic databases, including PubMed, Scopus, and Science Direct, to identify relevant studies on necroptosis assessment in AD. The search included publications from January 2015 to April 2025 and limited to articles published in the English language. The search strategy incorporated a combination of Medical Subject Headings (MeSH) and free-text keywords such as: "necroptosis", "RIPK1", "RIPK3", "MLKL", "pMLKL", "necroptosis inhibitors", "Alzheimer’s disease", "neurodegeneration", "tau pathology", and "Aβ deposition". Boolean operators (AND, OR) were used to combine terms effectively.

**Fig. 2:**
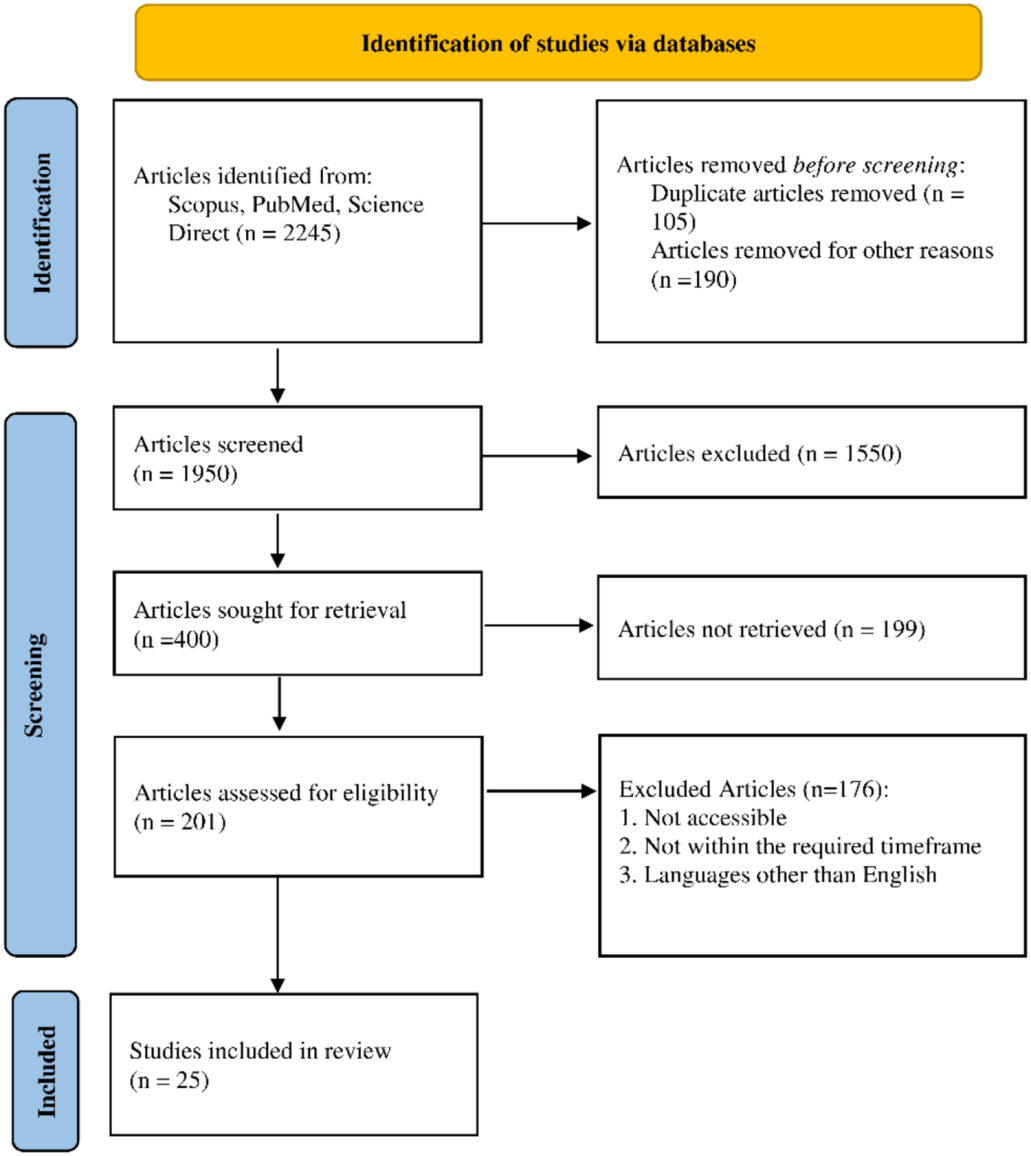
PRISMA Analysis

The study selection was restricted to original research and review articles that addressed the role of necroptosis in AD. To ensure thoroughness, gray literature was also reviewed using sources such as Google Scholar, individual journal websites, and manual searches of reference lists to retrieve additional relevant articles and clarify ambiguous data. This systematic review was conducted in accordance with the Preferred Reporting Items for Systematic Reviews (PRISMA). The process of study selection, including identification, screening, eligibility, and inclusion, is depicted in the PRISMA flow diagram below (Fig. 2).

The inclusion and exclusion criteria for the study are as

Inclusion criteria:

- Original research and review articles covering in vitro, in vivo, ex vivo, or clinical studies.
- Examination or discussion of necroptosis markers, including RIPK1, RIPK3, MLKL, or phosphorylated MLKL.
- Articles published between 2015 and 2025.
- Peer-reviewed publications in English (or with an available English translation).
- Studies analyzing necroptosis in association with AD pathologies, including Aβ deposition, tau phosphorylation, or neuroinflammation.

Exclusion criteria:

- Editorials, commentaries, or letters lacking original data or in-depth analysis.
- Research omitting necroptosis markers (RIPK1, RIPK3, MLKL, or pMLKL).
- Publications outside the 2015–2025 timeframe.
- Non-English articles without an official translation.

### Study Selection Process

The study selection process began with a careful review of all identified article titles. Initial exclusions were made for duplicate entries across the three primary databases (PubMed, Scopus, and ScienceDirect) as well as articles that failed to meet the predefined eligibility criteria. These included studies published outside the 2015–2025 timeframe, those not focused on necroptosis or AD, and publications in languages other than English. Following duplicate removal, the remaining articles underwent abstract screening to assess their relevance based on inclusion criteria. Priority was given to studies examining necroptosis markers (RIPK1, RIPK3, MLKL) and their association with AD pathology, including neuroinflammation, tau hyperphosphorylation, and amyloid-beta accumulation. Articles that did not meet these criteria were excluded from further analysis.

The final stage of the review involved a detailed full-text assessment of the remaining articles. Each study’s methodology was critically examined to determine its relevance and appropriateness for evaluating necroptosis in AD. A total of 25 rigorously selected studies were included in the synthesis, all of which explored key necroptotic markers and their contribution to AD pathogenesis. Although the review process was comprehensive, certain limitations remain. Restricting the search to English language publications may have introduced language bias, potentially omitting relevant non-English studies. Additionally, significant heterogeneity in study designs and inconsistencies in methodological reporting limited the feasibility of conducting a quantitative synthesis. Nevertheless, the study selection process, as illustrated in the PRISMA flow diagram (Fig. 2), was carried out with strict adherence to predefined inclusion criteria, ensuring that only methodologically robust and scientifically relevant research contributed to our understanding of necroptosis in AD.

### Necroptosis: Mechanisms and Key Molecular Players

#### Key Players of Necroptosis

Necroptosis is closely regulated by a group of signaling proteins, of which RIPK1, RIPK3, and MLKL are the key components of necroptotic machinery (Seo et al. 2021). RIPK1 is a pleiotropic protein that functions as an essential molecular switch between cell survival, apoptosis, and necroptosis. Structurally, RIPK1 has both a kinase domain and a RIP homotypic interaction motif (RHIM), allowing it to bind to other RHIM-containing proteins (Du and Wang 2024). In necroptotic situations, for example, when caspase-8 is inhibited, RIPK1 changes from survival promotion to necroptosis initiation (Orning and Lien 2021). This transition occurs by binding and recruiting RIPK3, creating the necrosome, a signaling platform needed for the downstream activation of the pathway. The kinase activity of RIPK1 plays an indispensable role in initiating necroptosis by mediating the phosphorylation of RIPK3, which in turn promotes its oligomerization and autophosphorylation (Seo et al. 2021).

RIPK3 is a serine/threonine kinase that serves as a key mediator of necroptosis. Upon necrosome formation, RIPK3 becomes activated and phosphorylates MLKL, the terminal effector of necroptosis (Morgan and Kim 2022). Apart from its kinase activity, RIPK3 also contributes as a signaling center through its interaction with metabolic enzymes and regulation of inflammatory responses (Riebeling et al. 2022). Activation of RIPK3 not only activates and enhances cell membrane breakdown via MLKL but also increases cytokine production, associating necroptosis with inflammation (Ermine et al. 2022). So, RIPK3’s function is particularly important in regulating the balance between pathological and protective inflammation, and its dysregulation is more and more linked with a variety of neurodegenerative and inflammatory diseases (Ke et al. 2023).

MLKL, the final effector of necroptosis, is a pseudokinase that consists of a four-helical bundle domain, a brace region, and a kinase-like domain (Chen et al. 2022a, b, c, d). Upon phosphorylation of RIPK3, MLKL undergoes conformational rearrangements that permit its oligomerization and subsequent translocation to the plasma membrane (Liccardi and Annibaldi 2023). At the membrane, MLKL disrupts cellular homeostasis by compromising membrane integrity, inducing ionic imbalance, promoting cell swelling, and ultimately causing membrane rupture (Chen et al. 2019). This membrane rupture leads to the passive release of DAMPs from the lysed cells, which in turn provokes a strong immune response, reinforcing the dual role of necroptosis as both a form of cell death and a contributor to sustained inflammation (Mázló et al. 2022).

#### Mechanistic of Necroptosis

**Fig. 3:**
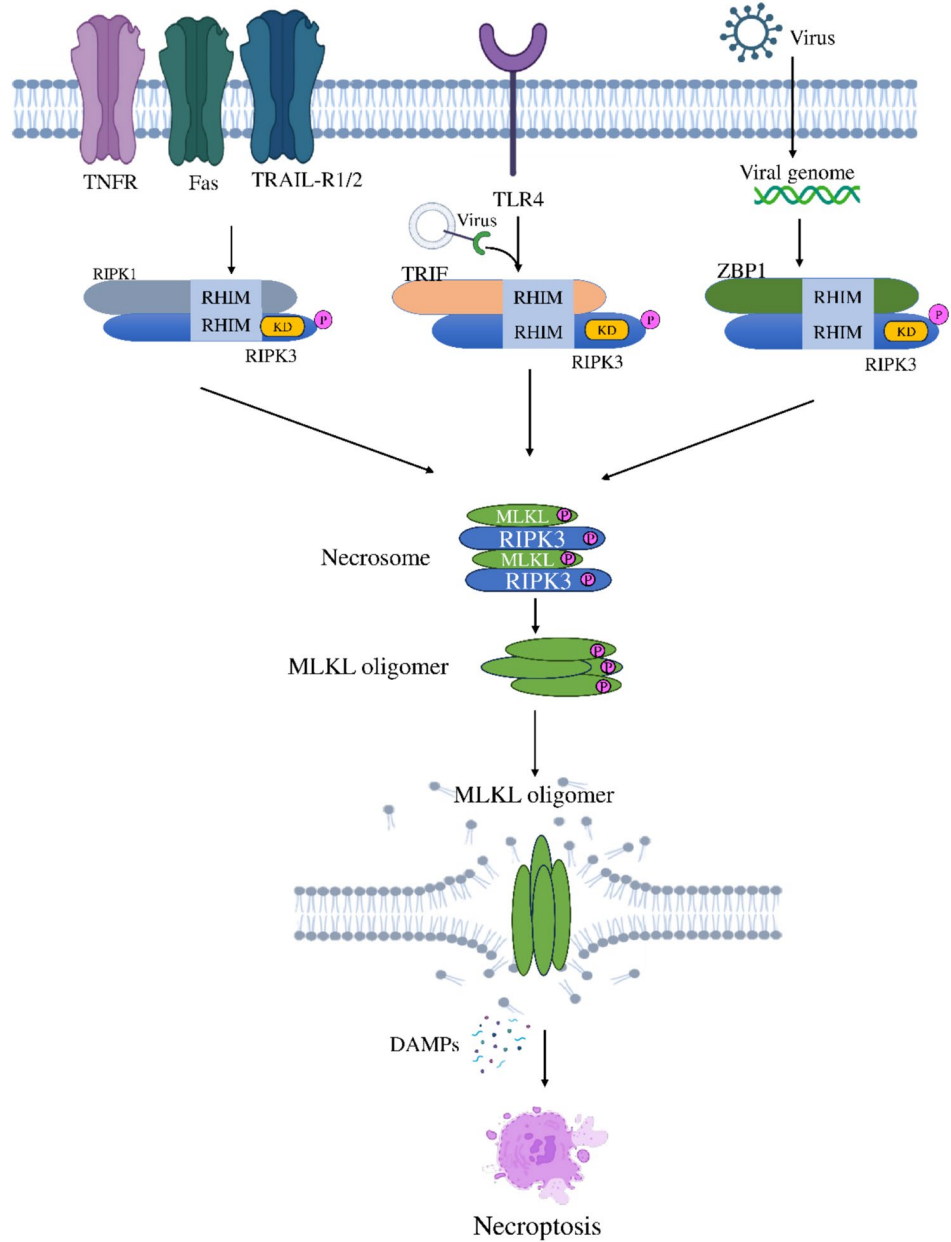
Different pathogenetic mechanisms of Necroptosis Signaling and key molecules involved

Several forms of signaling pathways by which necroptosis is activated, each of which involves different receptor-ligand interactions and molecular adaptors, but all converging on a central axis involving RIPK1, RIPK3, and MLKL. These include pathways mediated by TNF-α, TRAIL, Toll-like receptors (TLR3/4), Interferons (IFNs), Z-DNA-binding protein 1 (ZBP1/DAI), and the cGAS-STING axis. Figure 3 represents the various mechanistic pathways of necroptosis activation.

Figure illustrates diverse signaling pathways initiating necroptosis, a regulated form of cell death. Highlighting the key receptor-ligand interactions via TNFR, Fas, TRAIL-R1/2, TLR4, and ZBP1, and their downstream adaptors like RIPK1 and TRIF. The pathways converge on RIPK3 and MLKL activation, leading to necrosome formation and MLKL oligomerization. Ultimately, disruption of the cell membrane, releases damage-associated molecular patterns, resulting the inflammatory necroptotic cell death.

### TNF-α Mediated Necroptosis Pathway

The tumor necrosis factor receptor pathway is activated mainly by TNF-α, which binds to its respective receptors, TNFR1 and TNFR2, and a signaling cascade is initiated that decides the fate of the cell survival, apoptosis, or necroptosis (Sharma et al. 2021). On binding with TNF-α, it leads to the trimerization of the receptor and formation of Complex I. This receptor-bound signaling complex is made up of TNFR1-associated death domain protein (TRADD), receptor-interacting protein kinase 1(RIPK1), TNF-receptor-associated factor 2 (TRAF2), and E3 ubiquitin ligases such as cellular inhibitor of apoptosis proteins (cIAP1/2), in addition to the linear ubiquitin chain assembly complex (Manohar 2024). Ubiquitination of RIPK1, specifically by K63 ubiquitination by cIAP1/2, increases the activation of TAK1 through TAB2/TAB3, leading to NF-κB and MAPK activation, which is responsible for the induction of pro-inflammatory and pro-survival genes (Huang et al. 2025). In addition, LUBAC complex enhances NF-κB signaling through M1-type linear ubiquitination of RPK1 and TNFR1, thus increasing inflammatory responses by recruiting the IKK complex (IKKα, IKKβ, and NEMO/IKKγ). In general, regulation of RIPK1 is via phosphorylation by TAK1, IKKα/β, MK2, and TBK1, which is responsible for inhibiting excessive cellular death (Duan et al. 2022; Sasaki and Iwai 2023).

If conditions are conducive to cell death, deubiquitination of RIPK1 by CYLD (cylindromatosis) results in Complex I destabilization and the generation of Complex IIa, which consists of TRADD, FADD (Fas-associated death domain protein), RIPK1, and pro-caspase-8 (Engin 2021). Caspase-8 activation triggers apoptosis. But if caspase-8 activity is inhibited, RIPK1 binds to RIPK3 via their RHIM (RIP homotypic interaction motif) domains, resulting in the assembly of Complex IIb (the necrosome) (Orning and Lien 2021). Within this complex, RIPK1 is auto-phosphorylated (Ser14/15, Ser20, Ser161/166) and associates with RIPK3 through their RHIM domains, resulting in RIPK3 phosphorylation and activation (Ser227). Activated RIPK3 phosphorylates MLKL(Thr357/Ser358), resulting in oligomerization of MLKL and translocation to the plasma membrane, where it disrupts the membrane integrity, resulting in ion influx, cell swelling, and rupture (Chaouhan et al. 2022). This results in the release of damage-associated molecular patterns (DAMPs) such as ATP, HMGB1, and mitochondrial DNA, activating immune cells and propagating inflammation (Klegeris 2021). Although RIPK1 is an important regulator of TNF-α-induced necroptosis, necroptosis also proceeds independently of RIPK1 through TLR3/TLR4 activation and viral infection, where RHIM-containing proteins such as TRIF and ZBP1 directly activate RIPK3 (Udawatte and Rothman 2021).

### TNF-Related Apoptosis-Inducing Ligand (TRAIL) Pathway

The TRAIL pathway works similarly but is induced through the binding of TRAIL to death receptors (DR4 and DR5), which are members of the TNFR superfamily (Moriwaki et al. 2021). TRAIL binding causes DR4/DR5 trimerization, subsequent recruitment of FADD and pro-caspase-8, and formation of the death-inducing signaling complex (DISC). Under healthy circumstances, caspase-8 is activated and cleaves downstream caspases, initiating apoptosis. But if caspase-8 is blocked, TRAIL signaling can change direction to necroptosis (Pimentel et al. 2023). Further, RIPK1 and RIPK3 comprise the necrosome, with phosphorylation of MLKL resulting in the execution of necroptotic cell death. Nevertheless, resistance mechanisms, including caspase-8 inhibition or FLIP (FLICE-like inhibitory protein) expression, may redirect TRAIL signaling toward necroptosis rather than apoptosis (Engin 2021).

### Toll-Like Receptors (TLRs) Mediated Necroptosis Pathway

Aside from TNF signaling, Toll-like receptors are also essential in necroptosis, especially TLR3 and TLR4 (Wicherska-Pawłowska et al. 2021). These receptors are pattern recognition receptors (PRRs), recognizing microbial components like viral RNA (TLR3) and bacterial lipopolysaccharides (TLR4) (Li and Wu 2021). These receptors, upon binding to their ligands, bring in the adaptor proteins to facilitate immune signaling, such as Toll/IL-1 receptor (TIR) domain-containing adaptors. TLR3 signals solely through the TIR-domain-containing adaptor-inducing interferon-β/TIR-domain-containing adapter molecule-1 (TRIF/TICAM-1) adaptor, while TLR4 can use both TRIF and MyD88 (Manik et al. 2025). Necroptosis occurs in TLR-mediated signaling when caspase-8 activity is suppressed. For example, LPS-induced necroptosis in human macrophages happens when caspase-8 inhibition hinders apoptosis, initiating RIPK3-dependent MLKL activation. (Kim et al. 2022). Similarly, synthetic dsRNA analog poly (I: C) triggers apoptosis in Jurkat cells upon combination with IFN-γ, but when caspase-8 or FADD is absent, necroptosis is triggered instead (Molyer Yildirir 2024). These suggest that TLR3 and TLR4 pathways can start necroptosis, although precise regulatory mechanisms remain under investigation.

### Interferon (IFN)-Induced Necroptosis

Interferons are also known to induce necroptosis by distinct signaling pathways. IFN-induced necroptosis is dependent on Janus Kinase-1 (JAK1) and signal transducer and activator of transcription 1 (STAT1)-mediated transcription, which enables the assembly of a RIPK1-RIPK3 complex (Liu et al. 2024). The results underscore the intricate crosstalk among various receptors and cellular sensors in the control of necroptotic cell death. While each of the death receptors, TLRs, and IFN receptors participates in the initiation of necroptosis, it is via distinctive interactions that involve RHIM domain-containing proteins, including RIPK1, RIPK3, TRIF, and ZBP1 (Peng 2024).

### ZBP1/DAI-Induced Necroptosis

Z-DNA-binding protein 1 (ZBP1) or DNA-dependent activator of IFN-regulatory factors (DAI) is another mechanism that triggers necroptosis. ZBP1 is a cytoplasmic receptor of double-stranded nucleic acids and is a critical component of viral immunity and necroptosis control (Chen et al. 2022a, b, c, d). In contrast to TNF and TLR pathways, which are dependent on extracellular ligand-receptor interactions, ZBP1 binds to cytoplasmic nucleic acids, specifically Z-form DNA and RNA, commonly linked to viral replication (Zhan et al. 2024). In response to viral or endogenous Z-form nucleic acid recognition, ZBP1 oligomerizes, which allows it to directly recruit RIPK3 by its RHIM domain-containing regions. In cases where caspase-8 is inhibited or deficient, ZBp1-mediated necroptosis occurs as RIPK3 phosphorylates MLKL and induces membrane disruption and inflammatory cell death (Mishra et al. 2025). Most viruses contain caspase-8 inhibitors within their genome, promoting cell death toward necroptosis as an alternative cell death mechanism to counteract viral immune evasion.

### cGAS-STING Pathway Mediated Necroptosis

The cGAS-STING pathway is important in necroptosis as it senses cytosolic DNA and activates downstream signaling, resulting in regulated necrotic cell death. When double-stranded DNA derived from pathogens, dying cells, or mitochondrial leakage enters the cytoplasm, it is sensed by cyclic GMP-AMP synthase (cGAS) (Zheng et al. 2023). This activation leads to the synthesis of cyclic GMP-AMP (cGAMP), a second messenger that binds to STING (stimulator of interferon genes), a transmembrane protein located on the endoplasmic reticulum (ER) (Su et al. 2022). Activated STING is subject to conformational changes and translocates to the Golgi apparatus, recruiting TANK-binding kinase 1 (TBK1) there. TBK1 phosphorylates interferon regulatory factors (IRF3), which initiates the production of type I interferons (IFN-α/β) and pro-inflammatory cytokines. Further, STING directly binds to RIPK3, an essential necroptotic kinase, resulting in its autophosphorylation and activation. Activated RIPK3 phosphorylates MLKL, which oligomerizes and moves to the plasma membrane, resulting in membrane disruption, ion influx, and eventual cell rupture (Yuhan Chen et al. 2025). It reinforces inflammation by releasing DAMPs, propagating further immune responses.

Ultimately, necroptosis is a highly regulated, immune-driven form of cell death that integrates multiple pathways, including TNF, TLR, IFN, ZBP1, and cGAS-STING, to maintain host defense and eliminate infected or damaged cells. However, its dysregulation contributes to inflammatory and neurodegenerative diseases, highlighting the delicate balance between protective immunity and pathological cell loss. Understanding the precise molecular mechanisms governing necroptosis will be crucial for developing targeted therapies to modulate its effects in disease contexts. A summary of all the mechanisms is included in Table 1.

**Table 1:** Summary of mechanisms of necroptosis signaling

Pathway	Initiating Ligand	Key Receptor/Sensors	Adaptor/Signaling Molecules	Necrosome Components	Downstream effects
TNF-α pathway	Tumor Necrosis Factor-α (TNF-α)	TNFR1, TNFR2	TRADD, RIPK1, TRAF2, cIAP1/2, LUBAC, TAK1, IKKα/β, NF-κB	RIPK1, RIPK3, MLKL (if caspase-8 inhibited)	Cell membrane rupture, DAMPs release (ATP, HMGB1, mtDNA), inflammation
TRAIL pathway	TNF-related apoptosis-inducing ligand	DR4, DR5 (TNFR superfamily)	FADD, pro-caspase-8	RIPK1, RIPK3, MLKL (if caspase-8 inhibited or FLIP expressed)	Membrane disruption, necroptotic death
TLR pathway	PAMPs (e.g., LPS, dsRNA)	TLR3, TLR4	TRIF (for TLR3/4), MyD88 (for TLR4), FADD	RIPK3, MLKL (if caspase-8 or FADD is deficient)	Inflammatory necroptosis, DAMPs release
IFN pathway	Type I Interferons (IFN-α, IFN-β)	IFN receptors	JAK1, STAT1	RIPK1, RIPK3, MLKL	Regulated necroptosis via transcriptional induction of RIP kinases
ZBP1 pathway	Z-form DNA/RNA (viral/endogenous)	Cytosolic nucleic acid sensor ZBP1	ZBP1 (directly senses Z-DNA/Z-RNA)	RIPK3, MLKL (if caspase-8 is deficient)	Membrane rupture and inflammatory death during viral infection
cGAS-STING pathway	Cytosolic dsDNA (mitochondrial/pathogen-derived)	Cytosolic DNA sensor cGAS, receptor STING	cGAS → cGAMP → STING → TBK1 → IRF3, STING directly binds RIPK3	RIPK3, MLKL	MLKL activation, membrane rupture, DAMPs release, type I IFN production, inflammation

### Evidence of Necroptosis in AD

Several cellular, animal, and human models have given robust evidence in favor of necroptosis. Strong evidence from human studies has unequivocally placed necroptosis as a pathological contributor in AD.

#### Postmortem Brain Tissue Studies

A key study by Caccamo et al. (2017) examined postmortem brain tissues of AD patients and reported elevated expression of RIPK1, RIPK3, MLKL, and pMLKL, particularly in the hippocampus and entorhinal cortex, which are the most affected areas commonly associated with cognitive decline in AD. Their findings suggested that necroptosis activation could serve as a key pathological feature of AD progression (Caccamo et al. 2017). Additionally, in the study by Koper et al. (2022), the researchers examined how Limbic-predominant age-related TDP-43 encephalopathy with neurocognitive impairment (LATE-NC) exacerbates GVD-mediated necroptosis in AD. They found that in human postmortem brain tissue from AD patients with LATE-NC, there were significantly higher levels of pMLKL-positive GVD lesions, alongside increased tau pathology and neuronal loss, particularly in the hippocampus. This suggests that LATE-NC intensifies necroptosis, worsening neuronal damage and cognitive decline in AD (Koper et al. 2022). Furthermore, in postmortem brain tissue from late-onset AD patients, Degterev et al. (2019) observed elevated RIPK1 expression at both mRNA and protein levels. This increase correlated with lower brain weight and advanced Braak stages. Additionally, RIPK1-regulated genes like Ch25h were upregulated in vulnerable brain regions, linking necroptosis and lipid metabolism to AD progression (Degterev et al. 2019). In addition, in postmortem brain samples from AD patients, Koper et al. (2024) observed increased pMLKL and activated RIPK1 expression in neurons from the hippocampus and cortex, confirming that necroptosis is also active in human AD pathology. These human findings strongly aligned with the in vivo data using a mouse model, supporting necroptosis as a conserved and targetable mechanism of neuronal death in AD (Koper et al. 2024). Another study by Verheijen et al. (2022) utilized iPSC-derived cortical neurons to investigate sporadic AD (sAD) and its relation to environmental factors. The authors compared transcriptomic changes in sAD iPSC-derived neurons to postmortem brain samples. They found similar alterations in AD-related processes, with iPSC-derived neurons revealing more differentially expressed genes. Exposure to Alzheimerogens like copper and fipronil sulfone affected genes related to lipid metabolism and immune responses, supporting the involvement of inflammation and potentially necroptosis in AD (Verheijen et al. 2022). Furthermore, another notable study by Park et al. (2021) demonstrated that diminished O-GlcNAcylation enhances RIPK3 activity in AD brains. They further identified that O-GlcNAc modification at serine 350 normally inhibits RIPK3 activity. Loss of this modification promotes MLKL phosphorylation, triggering necroptotic neuronal death, and restoration of O-GlcNAcylation suppresses necroptosis, whereas it shows protective action for neurons. So, this study links metabolic dysfunction to necroptosis in AD (Park et al. 2021). Additionally, Jayaraman et al. (2021) investigated the role of necroptosis in AD. They found that the TNF receptor 1 (TNFR1) signaling pathway is upregulated in neurons within the hippocampus of AD patients. This upregulation leads to the activation of RIPK1 and MLKL, key components of the necroptotic pathway. Additionally, the study observed a downregulation of components of the endosomal sorting complex required for transport III (ESCRT-III), which are crucial for membrane repair. This imbalance contributes to plasma membrane rupture, a hallmark of necroptosis, thereby promoting neuronal loss in AD (Jayaraman et al. 2021).

#### In Vivo Studies

To support human findings, Caccamo et al. (2017) employed two well-established transgenic mouse models of Alzheimer’s disease: APP/PS1 and 5xFAD mice, both of which harbor human familial AD mutations in the APP and PSEN1 genes. These were compared with age- and sex-matched non-transgenic (NonTg) littermate controls. They assessed necroptotic activation by quantifying RIPK1, MLKL, and pMLKL levels. While APP/PS1 mice did not show significant necroptotic marker elevation at 12 months, 5xFAD mice at 11 months exhibited markedly increased levels of RIPK1, MLKL, and pMLKL compared to controls, correlating with prominent neuronal loss. To test the functional role of necroptosis, they overexpressed a constitutively active MLKL variant (caMLKL) via AAV delivery into APP/PS1 and NonTg mice, creating four experimental groups: APP/PS1-GFP, APP/PS1-MLKL, NonTg-GFP, and NonTg-MLKL. Behavioral analysis using the Morris Water Maze revealed that APP/PS1-MLKL mice performed significantly worse than other groups, particularly in spatial memory retention and learning, despite no changes in Aβ or tau pathology. NeuN staining further confirmed that caMLKL induced greater neuronal loss in APP/PS1 mice than in NonTg mice, highlighting that APP/PS1 brains are more susceptible to necroptosis. These comparisons established that necroptosis exacerbates neurodegeneration and cognitive impairment in an AD context and that its activation is a driving mechanism of neuronal death (Caccamo et al. 2017). Further, another study by Degterev et al. (2019) used 11-month-old 5xFAD and APP/PS1 transgenic mice to examine the necroptotic activity. Western blot analysis revealed significantly increased expression of RIPK1, MLKL, and pMLKL in the 5xFAD mice, but not in age-matched APP/PS1 mice. To evaluate functional outcomes, 5xFAD mice were treated with Necrostatin-1 s (Nec-1 s) at 10 mg/kg intraperitoneally and 0.5 mg/mL in drinking water for 21 days. This treatment reduced neuronal death, evidenced by fewer Fluoro-Jade-positive neurons, and lowered pMLKL/MLKL ratios, suggesting inhibition of necroptosis. These results confirmed that RIPK1-dependent necroptosis drives neurodegeneration in the 5xFAD model, while also revealing that RIPK1 modulates genes related to disease-associated microglia like Cst7, Csf1, and Clec7a (Degterev et al. 2019). Further, Koper et al. (2024) investigated necroptosis in Alzheimer’s disease using APP/PS1 transgenic mice and TauP301S mice, both of which model amyloid and tau pathologies, respectively. The researchers found elevated levels of pMLKL, indicating active necroptosis in vulnerable brain regions. Genetic deletion of MLKL or pharmacological inhibition using the RIPK1 inhibitor Necrostatin-1 s rescued neuronal loss and restored cognitive function in both models. These findings provided robust in vivo evidence that necroptosis contributes to neurodegeneration in AD and that its inhibition can be neuroprotective (Koper et al. 2024). In another study, in an Aβ₁₋₄₂-injected mouse model of Alzheimer’s disease, Tu et al. (2020) demonstrated that treatment with EGb761 (a standardized Ginkgo biloba extract) significantly ameliorated necroptotic signaling. Mice treated with EGb761 exhibited reduced expression of RIP1, RIP3, and MLKL, as well as improved mitochondrial membrane potential and decreased ROS generation in hippocampal tissue. These findings indicated that EGb761 mitigates necroptosis by modulating RIP1-mediated mitochondrial dysfunction and oxidative stress (Tu et al. 2020). Furthermore, the study by Ma et al. (2021) primarily focused on in vivo models using young and aged SAMP8 mice. The researchers investigated the effects of Cornel Iridoid Glycoside (CIG) on AD-like pathologies and necroptosis through the RIPK1/MLKL pathway in these mice. The study focused entirely on in vivo experimentation, confirming that CIG alleviates AD-like pathology by suppressing the RIPK1/MLKL axis involved in necroptosis (Ma et al. 2021). Similarly, Pang et al. (2022) developed a novel App^NL−G−F^ knock-in rat model carrying three familial AD mutations with a humanized Aβ sequence. Unlike mice, these rats showed AD-like Aβ plaques, tau pathology, synaptic loss, cognitive deficits, and importantly, activation of necroptosis marked by increased RIPK1, RIPK3, and pMLKL in the hippocampus. This model uniquely mimics human AD neurodegeneration and necroptosis, making it a valuable tool for drug discovery (Pang et al. 2022). In line with this, Jayaraman et al. (2021) in their in vivo model, used APP/PS1 transgenic mice and observed that increased TNF-α levels in the hippocampus triggered neuroinflammation, which was associated with necroptosis. Specifically, they found that necroptotic cell death was prominent in the hippocampal neurons of AD mice, and that blocking TNF-α signaling helped reduce the neuroinflammatory response and necroptosis. This suggests significant role of TNF-α in mediating necroptosis in AD and highlights the potential of targeting this pathway for therapeutic intervention in AD (Jayaraman et al. 2021). Moreover, in vivo experiments with APP/PS1 transgenic mice, Salvadores et al. (2022) found that inhibition of RIPK1 using Nec-1 reduced microglial activation and neuroinflammation. This intervention led to a decrease in Aβ-induced neurodegeneration, highlighting the potential for RIPK1 as a therapeutic target in AD (Salvadores et al. 2022). The study by Yang et al. (2017) explored the role of necroptosis in AD using APP/PS1 transgenic mice as an in vivo model. They found that Necrostatin-1, a specific RIPK1 inhibitor, alleviated cognitive impairment and reduced amyloid-beta plaques and tau abnormalities in the mice. This suggests that inhibiting necroptosis can help mitigate the cognitive and pathological features of AD (Yang et al. 2017). Another study by Naderi et al. (2024) used an in vivo rat model to examine the effects of Aβ25 − 35-induced neurotoxicity in AD. The rats were treated with Necrostatin-1, a specific inhibitor of necroptosis, to investigate its neuroprotective potential. The researchers focused on the dentate gyrus (DG) granule cells, where they observed that Aβ25 − 35 caused significant electrophysiological changes, including a reduction in neuronal excitability and a decrease in voltage-gated calcium channel (Ca^2+^) currents. However, administration of Nec-1 nearly restored the normal excitability and Ca^2+^ currents in these neurons. This suggests that necroptosis plays a critical role in Aβ25 − 35-induced neurotoxicity, and inhibiting necroptosis could potentially protect against the electrophysiological alterations associated with AD (Naderi et al. 2024). In support of this, Zou et al. (2020), using APP/PS1 AD mouse models, the authors found that venous and capillary cerebral endothelial cells (ECs) were particularly vulnerable to necroptosis, with activation of RIPK1, RIPK3, and MLKL. A reduction in mNat1 (murine N-acetyltransferase 1) expression in these ECs sensitized them to necroptosis. Restoring mNat1 expression in AD mice reduced necroptosis, improved blood–brain barrier integrity, and alleviated cognitive dysfunction. This study emphasizes the role of endothelial necroptosis in AD and the potential therapeutic benefit of targeting mNat1 (Zou et al. 2020).

#### In Vivo Studies: Chemically Induced Models

Zhang and Niu (2024) in their studies using AD animal models induced by Al, observed similar results. Al exposure led to increased necroptosis in the brains of these animals, reflected by upregulated expression of necroptosis-related proteins. Administration of Nec-1 in these animals also reduced necroptosis and attenuated neuronal loss. Compared to apoptosis and autophagy inhibitors, Nec-1 showed more prominent effects in reducing cell death and protecting against Al-induced neurodegeneration, highlighting the significance of necroptosis as the dominant cell death pathway in AD associated with aluminum exposure (Zhang and Niu 2024). Other chemically induced models support this as well. Additionally, He et al. (2021) investigated the neuroprotective effects of Qiangji Decoction (QJD) in a D-galactose-induced mouse model mimicking aging-related AD. Chronic D-gal exposure caused cognitive impairment, hippocampal neurodegeneration, and neuroinflammation, accompanied by suppressed AMPK/SIRT1 signaling and activated NF-κB pathways. QJD treatment significantly improved learning and memory, reduced neuronal apoptosis, and downregulated pro-inflammatory markers (He et al. 2021). In a study by Nasseri et al. (2020), intracerebroventricular (ICV) injection of streptozotocin (STZ) in rats was used to model sporadic AD. This model induced cognitive deficits and hippocampal neuroinflammation, mimicking AD-like pathology. Notably, there was a significant upregulation of necrosome components RIP1, RIP3, and TNF-α, indicating activation of necroptosis in the hippocampus. Treatment with Apelin-13, a neuroprotective peptide, for 15 days post-STZ injection, led to marked reductions in these necroptotic markers and improved learning and memory in the Morris Water Maze (Nasseri et al. 2020). Lastly, in a study by Gao et al. (2022), necroptosis was identified as a key contributor to cognitive decline in a zebrafish model of AD induced by aluminum trichloride. Aluminum exposure led to memory deficits, reduced acetylcholine levels, and increased AD-related gene and protein expression. Treatment with Necrostatin-1, a necroptosis inhibitor targeting RIPK1, significantly improved learning and memory, restored acetylcholine levels, and altered necroptosis-related gene expression. These results suggest that necroptosis plays a crucial role in aluminum-induced AD pathology and that Nec-1 holds promise as a therapeutic agent (Gao et al. 2022).

#### In Vitro Studies

Cell culture studies have yielded detailed mechanistic insights into necroptosis. In SH-SY5Y neuroblastoma cells exposed to Aβ₁₋₄₂, Tu et al. (2020) observed that EGb761 pretreatment suppressed cell death by attenuating RIP1 expression, preserving mitochondrial function, and lowering intracellular ROS levels. This effect was comparable to that of the RIP1 inhibitor Necrostatin-1, suggesting that EGb761 protects neuronal cells via a RIP1-dependent mechanism of necroptosis inhibition (Tu et al. 2020). Additionally, in the study by Salvadores et al. (2022), in vitro experiments using primary microglial cultures demonstrated that Aβ oligomers activated microglia, leading to necroptosis via the RIPK1/RIPK3/MLKL signaling pathway. This activation triggered the release of pro-inflammatory cytokines, contributing to neurodegeneration in AD (Salvadores et al. 2022). Furthermore, in vitro model, using primary neuronal cultures, Yang et al. (2017) showed that Nec-1 prevented Aβ-induced neuronal damage by inhibiting necroptosis. This further supports that necroptosis inhibition could be a potential therapeutic strategy for AD, particularly in protecting neurons from Aβ toxicity. (Yang et al. 2017). Jayaraman et al. (2021) in their in vitro model, used primary hippocampal neuronal cultures and exposed them to TNF-α and Aβ oligomers to mimic the neuroinflammatory environment of AD. They observed that TNF-α stimulation led to increased necroptosis in the neuronal cultures, which was also mitigated by blocking TNF receptor signaling. This provided further evidence that TNF-mediated neuroinflammation contributes to necroptosis in AD neurons, supporting the potential for TNF-α inhibitors as therapeutic agents in AD (Jayaraman et al. 2021). Another study by Yuan et al. (2020) explored the neuroprotective role of DHA in Aβ25-35-induced necroptosis in THP-1 monocytes. DHA pretreatment inhibited necroptotic markers (RIPK1, RIPK3, MLKL) and pro-inflammatory cytokines (TNF-α, IL-1β, IL-6), and blocked ERK1/2 activation. Co-treatment with DHA and Nec-1 synergistically suppressed RIPK3, indicating DHA acts via the RIPK1/RIPK3 pathway. Additionally, DHA restored Aβ-impaired monocyte migration, highlighting its therapeutic potential in AD-related necroptosis (Yuan et al. 2020). Finally, Naseri et al. (2022) investigated the neuroprotective effects of ghrelin in an amyloid-β_1-42_ (Aβ_1-42_)-induced rat model of AD. The study found that ghrelin improved memory performance and reduced the expression of pro-apoptotic proteins (Bax), necroptotic proteins (RIP1K and RIP3K), and the Bax/Bcl-2 ratio. Ghrelin also promoted autophagy by increasing Beclin-1 expression. The results suggest that ghrelin inhibits apoptosis and necroptosis while promoting autophagy, offering potential therapeutic benefits for AD (Naseri et al. 2022).

#### In Vivo and In Vitro - Combined Model

Bansal et al. (2025) explored the impact of Aβ on neuronal dysfunction in AD using the APP knock-in mouse model App^NL−G−F/NL−G−F^ (APP KI), which shows robust Aβ neuropathology. Utilizing both in vitro neuron-glia cocultures and in vivo brain tissue analyses, the author demonstrated that Aβ accumulation causes early disruption of the nuclear pore complex. This was evidenced by decreased expression of nucleoporins such as NUP98 and NUP107, along with impaired nucleocytoplasmic transport occurring even before the appearance of Aβ plaques. The disruption led to abnormal trafficking of proteins across the nuclear envelope and reduced levels of key transport proteins like importin-β1 and RanGAP1. Notably, the application of oligomeric Aβ alone was sufficient to induce these changes, while inhibiting Aβ production with a γ-secretase blocker partially restored nuclear integrity. Furthermore, neurons with compromised NPCs became more susceptible to TNF-α–triggered necroptosis, showing elevated activation of necroptotic markers RIPK3 and MLKL. Intervention through nuclear transport blockade or RIPK1 inhibition significantly reduced this cell death, suggesting that NPC impairment is an early and actionable event in Aβ-driven neurodegeneration in AD (Bansal et al. 2025). In another study by Chong Xu et al. (2021), the investigation of the intricate relationship between inflammation, necroptosis, and autophagy in the development of AD was conducted. Increased expression of necroptotic markers, such as phosphorylated RIPK1, RIPK3, and MLKL, was detected in both postmortem brain tissues of AD patients and APP/PS1 transgenic mouse models. These findings indicated enhanced activation of the necroptotic pathway. Additionally, experiments using primary cortical neurons exposed to TNF-α in vitro confirmed that TNF-α could induce necroptotic neuronal death via the RIPK1-RIPK3-MLKL signaling cascade. The study further revealed that RIPK1 interacts with p62/sequestosome 1(SQSTM1), which acts as a scaffold protein to facilitate necroptosis signaling. Importantly, the study identified a key regulatory role for UV Radiation Resistance Associated Gene (UVRAG), a protein involved in autophagy, in suppressing necroptosis. Under normal conditions, UVRAG interacts with the RIPK1-p62 complex to promote the degradation of RIPK1 through autophagy, thereby limiting necroptotic signaling, but in the context of AD, the expression of UVRAG was found to be significantly reduced. This reduction leads to impaired degradation of RIPK1, resulting in its accumulation and prolonged activation of necroptosis, which in turn contributes to neuronal loss (Xu et al. 2021).

#### Human Xenograft Model

In a groundbreaking study, Balusu et al. (2023) explored the role of the long noncoding RNA MEG3 in AD using an innovative human Rag2^−/−/^App^NL−G−F^ knock-in mouse, which is immunodeficient and carries three familial AD mutations. This setup allowed the human neurons to integrate into the mouse brain and be exposed to an AD-like pathological environment. Over time, the transplanted human neurons exhibited hallmark AD features, including phosphorylated tau (AT8-positive), granulovacuolar degeneration, and neuronal loss, while neurons transplanted into control mice did not show such alterations. Transcriptomic profiling of these xenografted human neurons revealed that MEG3 was among the most significantly upregulated genes in neurons exposed to the AD brain microenvironment. To confirm a functional role for MEG3, the researchers overexpressed MEG3 in human neurons in vitro, which led to robust activation of the necroptotic pathway, specifically marked by the activation of RIPK1, RIPK3, and MLKL. Conversely, knockdown of MEG3 in the xenografted human neurons prevented necroptosis, reduced neuronal loss, and improved cell survival within the AD mouse brain. Additionally, postmortem human AD brain samples revealed elevated MEG3 expression and colocalization with necroptosis markers, indicating that this mechanism is not only present in the model but also clinically relevant in human AD pathology. This study is pivotal as it establishes MEG3 as a human-specific driver of necroptosis in AD and underscores the utility of human neuron xenografts for uncovering disease mechanisms that are not observable in rodent neurons (Balusu et al. 2023).

#### *In Silico* Model

The article by Chen et al. (2022a, b, c, d) investigates the role of exercise in preventing necroptosis, a regulated form of cell death, in AD (Table 2). The study proposes that exercise may modulate exosomal miRNAs, particularly miR-215-5p, which can inhibit necroptosis-related genes like IDH1, BCL2L11, and SIRT1. The authors constructed a risk prediction model for AD using necroptosis-related genes and lncRNAs, achieving a high predictive power (AUC = 0.979). Through SCENIC analysis, key transcriptional regulators, CEBPB and GATA6, were identified as essential for the upregulation of miR-215-5p in skeletal muscle during exercise. These findings suggest that exercise can influence miRNA levels in circulating exosomes, which in turn prevent necroptosis and delay AD onset (Chen et al. 2022a, b, c, d).

**Table 2:** Summary of evidence studies of necroptosis in AD

Model	Species/source	Intervention	Markers Assessed	Key Takeaways	References
Human (Postmortem Study)	AD brain tissue	–	pRIPK1, pMLKL	Increased necroptotic markers in neurodegeneration-prone areas	Caccamo et al. (2017)
Human (Postmortem Study)	AD + LATE-NC brain tissue	–	pMLKL	pMLKL was increased in GVD, correlated with tau and neuron loss	Koper et al. (2022)
Human (Transcriptomic)	Transcriptomics and Human brain tissue	–	RIPK1, Ch25h	RIPK1 and lipid-related genes (e.g., Ch25h) were upregulated in AD brains	Degterev et al. (2019)
Human (Postmortem Study)	Cortex & hippocampus of the AD brain	–	pMLKL, RIPK1	Elevated pMLKL and RIPK1 in AD neurons; necroptosis linked with neurodegeneration	Koper et al. (2024)
In vitro (Human iPSC)	iPSC-derived human neurons	Nec-1, MLKL inhibitors	MLKL, RIPK3	Necroptotic activation reversed by RIPK1 and MLKL inhibition	Verheijen et al. (2022)
Human (Postmortem Study)	Human brain tissue analysis	O-GlcNAc modulation	RIPK3, MLKL, O-GlcNAc	Reduced O-GlcNAcylation activated RIPK3/MLKL; restoration blocked necroptosis	Park et al. (2021)
Human (Postmortem Study)	Human cortex	–	RIPK1, MLKL	RIPK1-MLKL upregulated, ESCRT-III repair system downregulated	Jayaraman et al. (2021)
Xenograft	Human neurons in App^NL−G−F^ mice	lncRNA MEG3 overexpression	RIPK1, RIPK3, MLKL	MEG3 induced necroptosis via RIPK1/RIPK3/MLKL	Balusu et al. (2023)
In vivo transgenic mice	5xFAD, APP/PS1 Mice	MLKL overexpression	MLKL, RIPK1	Overexpression of MLKL worsened memory and increased necroptosis and neuronal loss	Caccamo et al. (2017)
In vivo transgenic mice	APP/PS1, 5xFAD mice	Nec-1s	RIPK1	RIPK1 inhibition reduced Aβ pathology, restored microglial function, and improved synaptic health	Degterev et al. (2019)
In vivo Transgenic mice	APP/PS1, TauP301S mice	MLKL knockout, Nec-1s	RIPK1, pMLKL	MLKL KO and Nec-1 s reduced necroptosis, improved memory, and neuronal survival	Koper et al. (2024)
In vivo mouse model (Chemically Induced)	Aβ1–42-injected mice	EGb761 (Ginkgo biloba extract)	RIPK1, RIPK3, MLKL	Reduced necroptosis and oxidative stress; improved mitochondrial function and memory	Tu et al. (2020)
In vivo (aged mice)	SAMP8 mice	Cornel Iridoid Glycoside (CIG)	RIPK1, RIPK3, MLKL	CIG lowered necroptosis markers and enhanced memory	Ma et al. (2021)
In vivo transgenic mice	APP/PS1 mice	Anti-TNF therapy	RIPK1, MLKL	TNF-α induced necroptosis via RIPK1/MLKL; anti-TNF therapy reduced necroptosis and restored membrane repair capacity	Jayaraman et al. (2021)
In vivo Transgenic Mice	APPxTAU Mice	Nec-1	RIPK1, pMLKL	Nec-1 reduced microgliosis and neuronal damage	Salvadores et al. (2022)
In vivo Transgenic Mice	APP/PS1 mice	Nec-1	RIPK1, Aβ, Tau	Nec-1 restored memory, reduced Aβ and tau burden	Yang et al. (2017)
In vivo Transgenic mice	APP/PS1 mice	mNat1 restoration	RIPK1, mNat1	Loss of mNat1 triggered RIPK-mediated endothelial necroptosis, and restoration reversed changes	Zou et al. (2020)
In vivo Chemically Induced	AlCl₃-treated cortical neurons	Nec-1	RIPK1, RIPK3	Al increased necroptosis markers (RIPK1/RIPK3); Nec-1 reduced necroptosis and neuronal loss more effectively than autophagy or apoptosis inhibitors	Zhang and Niu (2024)
In vivo Chemically Induced	D-galactose-induced mice	Qiangji Decoction (QJD)	NF-κB, AMPK, SIRT1	Improved memory, reduced apoptosis, neuroinflammation, restored AMPK/SIRT1, and suppressed NF-κB	He et al. (2021)
In vivo Chemically Induced	STZ-ICV-injected rats	Apelin-13	RIPK1, RIPK3, TNF-α	Apelin-13 reversed necroptosis and improved memory	Nasseri et al. (2020)
In vivo transgenic rats	Aβ25–35-injected rats	Nec-1	Ca^2^⁺ current, excitability	Nec-1 normalized disrupted neuronal excitability and calcium current	Naderi et al. (2024)
In vivo Transgenic rats	APP^NL−G−F^ Rats	–	RIPK1, RIPK3, pMLKL	Demonstrated necroptosis pathway upregulation; model validated for AD necroptosis studies	Pang et al. (2022)
In vivo Chemically Induced	AlCl₃-induced zebrafish	Nec-1	RIPK1, RIPK3, MLKL	AlCl₃ impaired cognition and ACh; Nec-1 restored memory and reduced necroptosis gene expression	Gao et al. (2022)
In vivo & In vitro (Transgenic mice)	APP knock-in mouse model App^NL−G−F/NL−G−F^	γ-secretase inhibitor (DAPT), Nec-1s, nuclear transport blockers	RIPK1, RIPK3, MLKL, NUP98, NUP107	Aβ disrupted NPC function, impaired nucleocytoplasmic transport, and sensitized neurons to TNF-α–induced necroptosis; Nec-1 s and DAPT restored integrity	Bansal et al. (2025)
In vivo & In vitro (Transgenic mice)	APP/PS1 mice and primary neurons	RIPK1-p62-UVRAG interaction	RIPK1, p62, UVRAG	Necroptosis is linked with impaired autophagy through RIPK1 interactions	Xu et al. (2021)

### Potential Therapeutic Approach Targeting Necroptosis in AD

The therapeutic targeting of necroptosis in AD represents a promising disease-modifying approach due to the involvement of this pathway in neurodegeneration and neuroinflammation. One of the most investigated targets is RIPK1, a kinase that acts at the top of the necroptosis cascade and also regulates inflammation. Pharmacological inhibition of RIPK1 using molecules such as Necrostatin-1 and its more stable and selective analogue Nec-1s (7-Cl-O-Nec-1) has shown protective effects in multiple neurodegeneration models (Yuan et al. 2019). These inhibitors prevent the activation of downstream necroptotic mediators and simultaneously reduce the production of inflammatory cytokines by microglia. To overcome the limitations of early RIPK1 inhibitors, such as poor pharmacokinetics and limited brain penetration, novel compounds like GSK2982772 and DNL747 (SAR443060) have been developed (Chen et al. 2022a, b, c, d). Notably, DNL747 was optimized for CNS penetration and has progressed into early phase clinical trials for neuroinflammatory and neurodegenerative conditions, including ALS and AD (Hincelin‐Mery et al. 2024). Although DNL747 showed acceptable safety in Phase 1 trials, its clinical development was halted due to insufficient target engagement, highlighting the complexity of translating RIPK1 inhibition into effective CNS therapies. Nonetheless, continued optimization of RIPK1 inhibitors with improved BBB permeability and isoform selectivity remains a high priority (Vissers et al. 2022). Inhibiting RIPK1 could therefore mitigate both neuronal death and neuroinflammatory cascades that exacerbate synaptic dysfunction (Gong et al. 2024).

Further downstream, RIPK3 serves as a key kinase responsible for phosphorylating MLKL, the executioner protein of necroptosis; thus, targeting RIPK3 can effectively interrupt the progression of the necroptotic pathway. Experimental inhibitors like GSK’843, GSK’872, and HS-1371 have demonstrated RIPK3-specific inhibition, reducing neuronal death and glial activation in preclinical models (Zhou et al. 2019, 2024). While these inhibitors are still largely in experimental stages, they offer a strategic advantage by selectively blocking the necroptotic execution phase without disrupting other cellular processes. Unlike RIPK1, which also regulates apoptosis and inflammation, targeting RIPK3 may offer greater pathway specificity, but this comes at the cost of limited versatility in modulating upstream inflammatory responses.

At the terminal end of the pathway, MLKL oligomerization and translocation to the plasma membrane are essential for membrane disruption and cell lysis, and release of DAMPs, leading to neuroinflammation. Preventing this step could halt necroptosis even after upstream activation. Although specific MLKL inhibitors are still under development, small molecules such as necrosulfonamide (NSA) have been shown to bind human MLKL and inhibit its oligomerization and membrane insertion (Tang and Zhuang 2024). However, NSA is species-specific and nonfunctional in rodent models, limiting its utility in preclinical studies. Development of brain-penetrant, cross-species MLKL inhibitors remains an unmet need, though targeting MLKL is mechanistically attractive for preventing DAMP-induced inflammation and preserving cellular integrity.

Importantly, cell-type-specific roles of necroptosis are increasingly recognized in AD. Microglial necroptosis may exacerbate synaptic pruning and contribute to neuronal loss via cytokine-mediated toxicity, while astrocytic and neuronal necroptosis directly drive structural brain damage (Balestri et al. 2024). As such, timing and targeting strategies should be carefully refined. Early intervention, before widespread neuronal loss, could be critical for maximizing therapeutic benefit. Moreover, combining necroptosis inhibitors with conventional anti-amyloid or anti-tau therapies may produce synergistic effects by addressing multiple facets of AD pathology simultaneously (Tondo et al. 2024). This combinatorial approach could help overcome the limited efficacy of current monotherapies and improve patient outcomes. Thus, the modulation of necroptosis offers not only a mechanistically grounded therapeutic strategy but also the potential for personalized treatment approaches in AD.

## Conclusion and Future Directions

This systematic review consolidates the evidence identifying necroptosis as a mechanistically significant contributor to AD pathogenesis. Elevated expression and activation of the necroptotic mediators, i.e., RIPK1, RIPK3, and MLKL, have been consistently demonstrated across human postmortem tissues, iPSC-derived neurons, transgenic models, chemically induced models, and in vitro assays. These findings underscore necroptosis as an upstream and regulated driver of neurodegeneration, integrally associated with key pathological features of AD, including amyloid-beta deposition, tau hyperphosphorylation, synaptic loss, and chronic neuroinflammation. Preclinical studies employing pharmacological inhibitors of RIPK1 (e.g., Necrostatin-1, Nec-1 s) and MLKL (e.g., necrosulfonamide) have demonstrated encouraging results, including the preservation of neuronal viability, reduced neuroinflammation, and partial restoration of cognitive function. These findings suggest that targeting necroptosis may offer a novel therapeutic strategy that addresses both neuronal death and inflammatory cascades, potentially complementing or surpassing mono-targeted symptomatic treatments.

Despite these promising advances, the translation of necroptosis inhibition into clinical therapies remains in its early stages and faces several critical challenges. The development of brain-penetrant, isoform-selective, and metabolically stable inhibitors for RIPK1, RIPK3, or MLKL is a prerequisite for human application. Equally vital is the thorough assessment of their safety, pharmacokinetics, and clinical efficacy in well-controlled trials. Parallel efforts should also focus on the identification and longitudinal validation of necroptosis-associated biomarkers, such as phosphorylated MLKL or RIPK3, in cerebrospinal fluid and plasma, as studies have detected elevated levels of pMLKL in the CSF of AD patients, which suggests its potential as a diagnostic indicator but also as a predictor of therapeutic response and disease progression. Understanding the temporal dynamics of necroptosis activation across different stages of AD is also imperative. Early stage intervention may yield maximal therapeutic benefits by preventing irreversible neuronal damage and AD. Moreover, exploring the complex intersection between necroptosis and other regulated cell death pathways, including apoptosis, ferroptosis, and pyroptosis, may also uncover novel nodes of convergence and provide a rationale for combinatorial therapeutic strategies. Additionally, research into the genetic, epigenetic, and transcriptomic regulation of necroptotic machinery could enhance our understanding of individual susceptibility to AD and aid in tailoring personalized therapeutic approaches.

In conclusion, necroptosis represents a biologically robust and increasingly validated pathway contributing to AD pathology. While its therapeutic targeting holds considerable evidence, substantial preclinical and clinical validation is required to establish its role as a disease-modifying intervention. Continued research into the regulation, modulation, and translational application of necroptosis could mark a transformative step forward in the development of effective interventions for AD, shifting the focus from symptomatic relief to mechanistic disease reversal and neuronal preservation.

## Data Availability

No datasets were generated or analyzed during the current study.
